# Comparison of the initial and residual speed of *Ixodes scapularis* kill on dogs treated with a single dose of Bravecto^®^ Chew (25 mg/kg fluralaner) or Simparica TRIO^®^ (1.2 mg/kg sarolaner, 24 µg/kg moxidectin, 5 mg/kg pyrantel)

**DOI:** 10.1186/s13071-023-05946-3

**Published:** 2023-11-27

**Authors:** Kathryn E. Reif, Naemi P. Bickmeier, Brian H. Herrin, Michael W. Dryden, Dorothy M. Normile, Jeba R. J. Jesudoss Chelladurai, Kamilyah R. Miller, Macy R. Flowers, Qing Kang

**Affiliations:** 1 Department of Diagnostic Medicine/Pathobiology, Kansas State University, Manhattan, KS 66506 USA; 2 Merck Animal Health, 2 Giralda Farms, Madison, NJ 07940 USA; 3 Department of Statistics, Kansas State University, Manhattan, KS 66506 USA

**Keywords:** Acaricide, Blacklegged tick, Canine, Deer tick, Ectoparasiticide, Isoxazoline, Prevention, Speed of kill, Tick, Tick control

## Abstract

**Background:**

Compliant ectoparasiticide product use is a comprehensive way to control ticks and reduce the risk of tick-borne pathogen transmission to dogs. Because the systemically acting isoxazoline ectoparasiticides require tick attachment for drug delivery, fast speed of kill is essential to minimize tick-borne pathogen transmission risk.

**Methods:**

Dogs of satisfactory tick-carrying capacity were randomly allocated to treatment groups and administered, per label instructions, Bravecto^®^ Chews (minimum 25 mg/kg fluralaner), Simparica TRIO^®^ (minimum 1.2 mg/kg sarolaner, 24 µg/kg moxidectin, 5 mg/kg pyrantel), or no treatment. Dogs were infested with approximately 50 unfed adult (35 female, 15 male) *Ixodes scapularis* on Day -2, 21 and 28. Live tick counts were performed at 4, 8, 12 and 24 h post-treatment (Day 0) and post-infestation on Day 21 and 28. Tick control efficacy was determined by comparing live tick means for each product-treated group to the untreated control group and each other at all time points using a linear mixed model. The percent of dogs free of live ticks was analyzed using the Fisher’s exact test for treatment group comparison.

**Results:**

The untreated control group maintained adequate tick infestations throughout the study. Using geometric means, an existing *I. scapularis* infestation was controlled by 99.7% and 93.0% 12 h post-treatment and by 100% and 99.5% 24 h post-treatment, for Bravecto^®^ and Simparica TRIO^®^-treated dogs, respectively. *Ixodes scapularis* infestations were controlled more quickly for Bravecto^®^- compared to Simparica TRIO^®^-treated dogs on Day 21 at 8 h (efficacy 74.0% vs. 0.0%, *p* = 0.003) and 12 h (efficacy 99.2% vs. 39.4%, *p* < 0.001) post-infestation and Day 28 at 8 h (efficacy 92.2% vs. 0.0%, *p* < 0.001) and 12 h (efficacy 99.6% vs. 27.7%, *p* < 0.001) post-infestation. On Day 28 post-treatment, the efficacy of Bravecto^®^ and Simparica TRIO^®^ to control a new *I. scapularis* infestation was 100% and 96.6%, respectively, by 24 h post-infestation. Of product-treated dogs, 100% of Bravecto^®^-treated dogs were free of live ticks by 24 h post-treatment or post-infestation. No treatment-related adverse reactions occurred during the study.

**Conclusions:**

*Ixodes scapularis* infestations are controlled more quickly 21 and 28 days post-treatment for dogs administered a single dose of Bravecto^®^ compared to dogs administered a single dose of Simparica TRIO^®^.

**Graphical Abstract:**

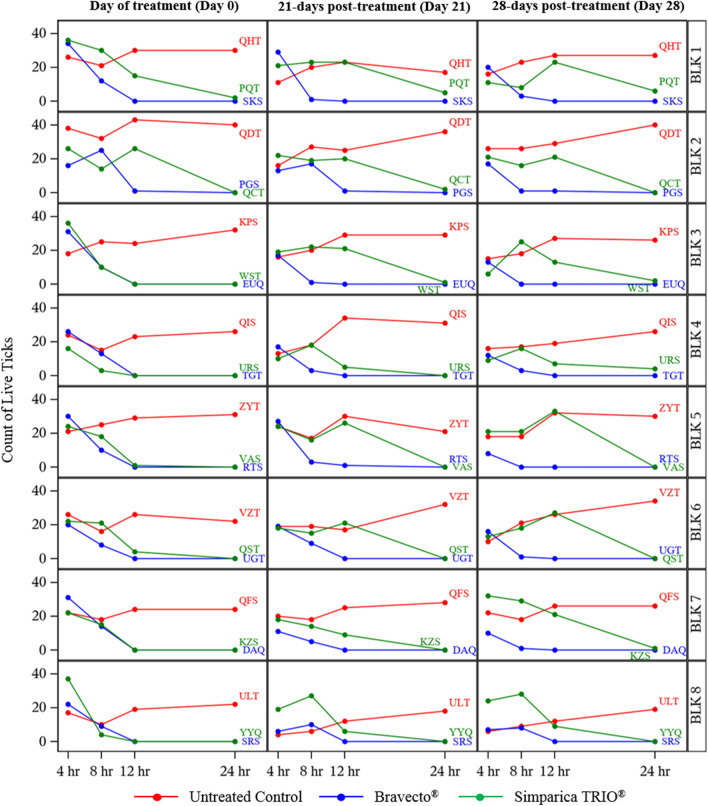

**Supplementary Information:**

The online version contains supplementary material available at 10.1186/s13071-023-05946-3.

## Background

Control of tick infestations and the various bacterial, viral and protozoal pathogens they transmit to dogs is of critical importance to canine health [1, 2]. The most comprehensive way to control ticks on dogs and reduce the risk of tick-borne pathogen transmission is compliant use of ectoparasiticide control products [3, 4]. Administration of ectoparasiticide products to dogs should start as early as the product labels allow and be maintained throughout their life. In many areas of the US, ticks are active year-long, necessitating the compliant use of an ectoparasiticide product year-round to protect dogs [3]. Seasonal approaches to parasite control, particularly tick infestations, is difficult because of year-to-year temperature fluctuations and expanding tick populations [4, 5]. In North America, intensification of resident tick populations and expansion of tick species into geographic regions they previously did not inhabit or repopulation into areas where they had been extirpated are well documented [1, 5–8]. A variety of ecological factors are proposed for the recent increase in tick numbers and range expansion, including increase in favorable habitat due to reforestation, changes in agricultural land use patterns, climate fluctuations and the remarkable increase in white-tailed deer populations [1, 6–8]. In addition to several native tick species that regularly parasitize dogs, a recently introduced tick species that also infests dogs, *Haemaphysalis longicornis*, is rapidly spreading across eastern and central US [9–11].

One tick species that has seen great expansion and is of upmost importance to canine health is *Ixodes scapularis* [5, 12–15]. This tick species is the vector of *Borrelia burgdorferi* and *Anaplasma phagocytophilum* to dogs in central and eastern North America. Tick control products administered to dogs must be effective against already attached ticks and, even more importantly, need to provide excellent and rapid tick killing activity against newly acquired ticks. The more rapidly a newly acquired tick can be killed, the less time the attaching tick has to transmit a pathogenic agent to a dog. Therefore, a product’s residual-speed of tick kill is important in the clinical success of an acaricide. Historically, numerous topical modalities have been used for tick control including collars, sprays, shampoos and spot-ons [16]. More recently, a new family of systemically acting compounds called isoxazolines has offered the option for oral and transdermal administration of drugs effective against fleas and ticks as well as various other external parasites [16, 17].

Members of the isoxazoline drug class (e.g. fluralaner, sarolaner, afoxolaner, lotilaner) are systemically acting acaricides and insecticides that antagonize GABA- and glutamate-gated chloride channel receptors that result in paralysis and eventual death of ticks and other blood-feeding ectoparasites that commonly parasitize dogs [17, 18]. Fluralaner and sarolaner are two isoxazoline drugs that are formulated in Bravecto^®^ and Simparica^®^ products, respectively. Bravecto^®^ (25 mg/kg fluralaner) Chews for dogs is the only extended duration (up to 12 weeks) ectoparasiticide approved in the US for control of fleas and five tick species, including *Amblyomma americanum, Rhipicephalus sanguineus, Dermacentor variabilis, Ixodes scapularis* and, most recently, *H. longicornis*. While detailed efficacy and speed of kill studies have been published for fluralaner against *Ixodes ricinus* [19, 20], the only peer-reviewed published “efficacy” data for fluralaner against *I. scapularis* infesting dogs is from a study where the weights of ticks and their coxal index post-feeding on treated dogs were evaluated [21]. In that report, 100% of the ticks were demonstrated dead 12 h after infestation at 4 weeks post-treatment [21]; however, no temporal speed of tick kill data is provided. Simparica TRIO^®^ (1.2 mg/kg sarolaner, 24 µg/kg moxidectin, 5 mg/kg pyrantel) is a monthly parasite control combination product that is approved in the US for the control of fleas, five tick species (*A. americanum*, *Amblyomma maculatum*, *D. variabilis*, *I. scapularis*, *R. sanguineus*), heartworm disease (*Dirofilaria immitis*) roundworms (*Toxocara canis, T. leonina*) and hookworms (*Ancylostoma caninum, Uncinaria stenocephala*). Of note, the minimum dose of sarolaner, the tick killing compound, in Simparica TRIO^®^ (1.2 mg/kg) was reduced by 40% compared to the amount of sarolaner in the stand-alone product Simparica^®^ (2.0 mg/kg).

The objective of this study was to determine the initial and residual speed of tick kill of a single treatment of, orally administered, fluralaner chewable (Bravecto^®^ Chews) against *I. scapularis* infesting dogs. Furthermore, the efficacy of a single treatment of fluralaner to control *I. scapularis* infestation in dogs was compared against the efficacy of a single treatment of a newly released isoxazoline combo-product containing sarolaner-moxidectin-pyrantel (Simparica TRIO^®^).

## Methods

### Compliance

This study was conducted in accordance with the World Association for the Advancement of Veterinary Parasitology (WAAVP) guidelines for evaluating the efficacy of parasiticides for the treatment, prevention and control of flea and tick infestations of dogs and cats [22] and the Good Clinical Practice guidelines [23]. This study was conducted as a masked, randomized, complete block design positive- and negative-controlled laboratory efficacy study. All personnel involved in animal observations, infestation procedures, tick count and assessment procedures were masked to the treatment status of each dog. Personnel conducting tick infestations and tick counts wore personal protective equipment to avoid skin contact with dogs. Gloves were changed after performing tick counts on each dog within a group, and gowns and gloves were changed between performing tick counts on dogs in different groups to ensure no possible transfer of any active ingredient. Personnel involved in treatment administration were not involved in conducting or recording tick counts or dog health assessments. All animal work was conducted in full compliance with an approved Institutional Animal Care and Use Committee Protocol (IACUC Protocol 4534) on file with the University Research Compliance Office at Kansas State University in Manhattan, KS.

### Animals and allocation

Twenty-four purpose-bred beagles (> 6 months of age, 5.2–8.5 kg, 13F:11M) that had demonstrated satisfactory tick-carrying capacity (> 25% of test infestation recovered) were selected for inclusion in the study. Enrolled dogs were ranked in order from highest to lowest live tick count and then blocked into eight groups of three based on descending live tick count. Within each block, dogs were randomly assigned to one of three groups using the randomization function on a spreadsheet program (Microsoft Excel 2019, Redmond, WA). Dogs were housed individually in concrete runs and did not have any contact with each other. Dogs housed in runs in the same room could hear one another and see one another when moved for run cleaning. Dogs were fed a maintenance ration once daily and provided water ad libitum. Unless otherwise noted, general health observations were performed daily for all dogs.

### Tick infestations

Laboratory-reared, adult *I. scapularis* (Oklahoma State University, Stillwater, OK) were purchased and shipped from the vendor by overnight courier to Kansas State University. Before being placed on dogs, ticks were maintained in the laboratory for up to 2 weeks at room temperature and 92% to 94% relative humidity. Male and female ticks were maintained in separate containers until time of infestation. Tick infestations were conducted on awake dogs on Study Day -2, 21 and 28. At each infestation time point, each dog was infested with approximately 50 unfed adult *I. scapularis* (35 female, 15 male). To infest the dogs, the lid of the tick container was removed and a study personnel exhaled slowly over the top of the container to stimulate tick activity. Ticks were applied directly to the dog’s fur along the dorsal side of the head, neck and back. Within 5 min of the tick infestation, dogs were transferred to pet carriers for 4 h to restrict activity and facilitate tick attachment. Following this 4-h period, dogs were transferred back to their individual pens for the duration of the study. Pet carriers were inspected, and any ticks that failed to infest dogs were collected and disposed.

### Treatments

On Study Day 0 (i.e. day of treatment), dogs in Treatment Group 1 received no treatment (i.e. untreated controls); each dog in Treatment Group 2 received a fluralaner chewable tablet (Bravecto^®^ Chew, Merck Animal Health, Madison, NJ, USA) based on the dog’s individual body weight to achieve a minimum dose of 25 mg fluralaner/kg; each dog in Treatment Group 3 received a sarolaner-moxidectin-pyrantel chewable tablet (Simparica TRIO^®^, Zoetis Animal Health, Parsippany-Troy Hills, NJ) based on the dog’s individual body weight to achieve a minimum dose of 1.2 mg sarolaner/kg, 24 µg/kg moxidectin and 5.0 mg/kg pyrantel. Both the fluralaner and sarolaner combination products were administered per product label and approximately 30 min after the dogs’ morning meal. Dogs in the untreated control group were similarly handled but received only a small spoonful of wet dog food. Each dog was observed at 1, 2 and 4 h post-treatment administration to assess whether the chewable tablets were spit or vomited out and to monitor for any signs of adverse treatment reactions. No dog spit/vomited the chewable tablet, and no dog experienced any adverse treatment reaction.

### Tick counts

Tick counts for initial speed of tick kill analysis were conducted at 4, 8, 12 and 24 h (± 0.25 h) post-treatment. Tick counts for residual speed of tick kill analysis were conducted 4, 8, 12 and 24 h (± 0.25 h) post-infestation on Day 21 and Day 28. Dogs were placed on a stainless-steel examination table and visibly inspected for ticks. Tick counts were conducted by manually restraining and examining the entire body of each dog for the presence of attached and unattached ticks for up to 20 min. The examination procedure involved running a gloved finger and/or flea comb against the lay of the hair so the hair could be parted to visually inspect for ticks. The examination commenced on the dog’s head, proceeded to the back, lateral sides, abdomen, chest and legs and feet (with careful inspection between the toes). Each observed tick was determined to be male or female, live or dead, and attached or unattached. Ticks were left on the dog following the 4-, 8- and 12-h counts but were removed with forceps after the 24-h tick count. Consistent with WAAVP guidelines [22], a tick was classified as *live* if it was attached or unattached and had any observable leg movement in response to stimuli. A tick was classified as *dead* if it displayed no movement in response to stimuli (e.g. no leg motion when stimulated by breathing on or probing the tick with fingers or forceps). Moribund ticks that exhibited any leg movement were classified as *live*.

### Statistical analysis

Counts of live ticks were analyzed separately for each time point of every post-enrollment infestation, using a linear mixed model. Treatment group was the fixed effect, and block was the random effect. Error term variance was taken as heterogeneous regarding treatment group. For efficacy assessment based on geometric means, counts of live ticks were subjected to $\ln ({\text{count}} + 1)$ transformation before statistical modeling. Denote ${\text{LSM}}_{\ln } (i)$ as the corresponding least squares mean of treatment group *i*, efficacy of treatment group *i* relative to treatment group *j* was calculated as

$$1 - \frac{{\exp ({\text{LSM}}_{\ln } (i)) - 1}}{{\exp ({\text{LSM}}_{\ln } (j)) - 1}},$$

where $\exp ({\text{LSM}}_{\ln } (i)) - 1$ represents the model-based estimate of geometric mean for treatment group *i*. For efficacy assessment based on arithmetic means, counts of live ticks were modeled directly without any transformation. Denote ${\text{LSM}}(i)$ as the corresponding least squares mean of treatment group *i*. Efficacy of treatment group *i* relative to treatment group *j* was calculated as

$$1 - \frac{{{\text{LSM}}(i)}}{{{\text{LSM}}(j)}},$$

where ${\text{LSM}}(i)$ represents the model-based estimate of arithmetic mean for treatment group *i*. To assure convergence, variances for block and error term were bounded low by 10^–3^ for transformed counts and 10^–1^ for untransformed counts.

A tick-free dog was defined as a dog with no live ticks [i.e. an infested dog has one or more live tick(s)]. The percentage of tick-free dogs by treatment group was analyzed using the Fisher’s exact test for treatment group comparison at a given time when both groups were not 100% infested.

All tests were conducted at the 0.05 significance level. Pairwise comparisons were carried out using two-sided tests. No multiplicity adjustment was performed.

Statistical analyses were performed using Statistical Analysis Software (SAS version 9.4; Cary, NC) PROC MIXED.

## Results

### Product dosing and safety

Overall, dogs averaged 6.3 kg (Treatment Group 1—6.3 kg, Treatment Group 2—6.2 kg, Treatment Group 3—6.5 kg). Dogs in Treatment Group 2 received an average of 40.1 mg/kg fluralaner (range 29.6–48.3 mg/kg). Dogs in Treatment Group 3 received an average of 1.9 mg/kg sarolaner (range 1.6–2.3 mg/kg). No dog in either product-treated group had an adverse event immediately after treatment or within 1, 2 or 4 h post-treatment administration. No dog spit the chew out or required re-treatment. No serious adverse events were observed during the course of the study. One dog in the Treatment Group 2 (Study Day 12) and two dogs in Treatment Group 3 (Study Day 2 and 26) had soft stools/diarrhea of 24–48 h duration that resolved without treatment intervention.

### Tick counts and product efficacy

Results presented below are based on analyses using geometric means. Tables 1 and 2 provide data and analyses using both geometric and arithmetic means. Additional file 1: Figure S1 presents the individual dog live tick counts by dog block.

**Table 1:** Summary statistics for live tick numbers on dogs treated with Bravecto^®^ or Simparica TRIO^®^

Tick count time^1^	Treatment group	Day -2 infestation (treatment and tick counts on Day 0)						Day 21 infestation						Day 28 infestation					
		N	# of live ticks				# (%) tick-free dogs	N	# of live ticks				# (%) tick-free dogs	N	# of live ticks				# (%) tick-free dogs
			Geometric mean	Arithmetic mean	Min	Max			Geometric mean	Arithmetic mean	Min	Max			Geometric mean	Arithmetic mean	Min	Max	
4 h	Untreated control	8	23.3	24.0	17	38	0 (0.0%)	8	14.0	15.4	4	24	0 (0.0%)	8	15.0	16.1	6	26	0 (0.00)
	Bravecto^®^	8	25.5	26.3	16	34	0 (0.0%)	8	15.8	17.4	6	29	0 (0.0%)	8	12.2	12.9	7	20	0 (0.0%)
	Simparica TRIO^®^	8	26.4	27.4	16	37	0 (0.0%)	8	18.4	18.9	10	24	0 (0.0%)	8	15.2	17.1	6	32	0 (0.0%)
8 h	Untreated control	8	19.2	20.3	10	32	0 (0.0%)	8	17.0	18.1	6	27	0 (0.0%)	8	18.1	18.8	9	26	0 (0.0%)
	Bravecto^®^	8	11.9	12.6	8	25	0 (0.0%)	8	4.4	6.1	1	17	0 (0.0%)	8	1.4	2.1	0	8	2 (25.0%)
	Simparica TRIO^®^	8	11.7	14.4	3	30	0 (0.0%)	8	18.8	19.3	14	27	0 (0.0%)	8	18.9	20.1	8	29	0 (0.0%)
12 h	Untreated control	8	26.5	27.3	19	43	0 (0.0%)	8	23.3	24.4	12	34	0 (0.0%)	8	23.9	24.8	12	32	0 (0.0%)
	Bravecto	8	0.1	0.1	0	1	7 (87.5%)	8	0.2	0.3	0	1	6 (75.0%)	8	0.1	0.1	0	1	7 (87.5%)
	Simparica TRIO^®^	8	1.8	5.8	0	26	4 (50.0%)	8	14.1	16.4	5	26	0 (0.0%)	8	17.3	19.3	7	33	0 (0.0%)
24 h	Untreated control	8	27.8	28.4	22	40	0 (0.0%)	8	25.7	26.5	17	36	0 (0.0%)	8	27.9	28.5	19	40	0 (0.0%)
	Bravecto^®^	8	0.0	0.0	0	0	8 (100%)	8	0.0	0.0	0	0	8 (100%)	8	0.0	0.0	0	0	8 (100%)
	Simparica TRIO^®^	8	0.1	0.3	0	2	7 (87.5%)	8	0.6	1.0	0	5	5 (62.5%)	8	1.0	1.6	0	6	4 (50.0%)

^1^Tick count time posttreatment (Day 0) or postreinfestation (Day 21 or 28)

**Table 2:** Efficacy of Bravecto^®^ and Simparica TRIO^®^ to control adult *Ixodes scapularis* infestation upon treatment (Day 0) and at 21 and 28 days post-treatment

			Model ln(count + 1)					Model count directly				
				Compared to untreated control		Compared to Simparica TRIO^®^			Compared to untreated control		Compared to Simparica TRIO^®^	
Infestation day^1^	Tick count time^2^	Treatment	Geometric mean ± SE	Test statistic (DOF) *P* value	Efficacy (%)^3^	Test statistic (DOF) *P* value	Efficacy (%)^3^	Arithmetic mean ± SE	Test statistic (DOF) *P* value	Efficacy (%)^3^	Test statistic (DOF) *P* value	Efficacy (%)^3^
Day -2 (48 h prior to treatment)	4 h	Untreated control	23.3 ± 2.1					24.0 ± 2.3				
		Bravecto^®^	25.5 ± 2.4	0.7(13.8) 0.495	0	− 0.2(13.3) 0.822	3.2	26.3 ± 2.2	0.7(14.0) 0.497	0	− 0.3(13.3) 0.759	4.1
		Simparica TRIO^®^	26.4 ± 2.8	0.9(13.4) 0.392	0			27.4 ± 2.8	0.9(13.5) 0.371	0		
	8 h	Untreated control	19.2 ± 2.3					20.2 ± 2.2				
		Bravecto^®^	11.9 ± 1.7	− 4.1(7.4) 0.004	38.0	0.1(8.3) 0.951	0	12.6 ± 2.0	− 3.8(5.9) 0.009	37.7	− 0.5(7.9) 0.620	12.2
		Simparica TRIO^®^	11.7 ± 3.1	− 2.0(5.9) 0.096	39.0			14.4 ± 3.5	− 1.7(8.0) 0.138	29.0		
	12 h	Untreated control	26.5 ± 2.3					27.3 ± 2.5				
		Bravecto^®^	0.1 ± 0.1	− 63(7.2) < 0.001	99.7	− 2.3(7.0) 0.056	95.1	0.1 ± 0.2	− 11(7.0) < 0.001	99.5	− 1.7(7.0) 0.138	97.8
		Simparica TRIO^®^	1.8 ± 1.2	− 5.4(6.8) 0.001	93.0			5.7 ± 3.4	− 5.1(12.9) < 0.001	78.9		
	24 h	Untreated control	27.8 ± 2.1					28.4 ± 2.2				
		Bravecto^®^	− 0.0 ± 0.0	− 47(7.2) < 0.001	100	− 1.0(7.0) 0.351	100	0.0 ± 0.2	− 13(7.0) < 0.001	100	− 1.0(8.5) 0.366	100
		Simparica TRIO^®^	0.1 ± 0.2	− 21(10.4) < 0.001	99.5			0.3 ± 0.3	− 13(7.1) < 0.001	99.1		
Day 21 (21 days post-treatment)	4 h	Untreated control	14.0 ± 2.6					15.4 ± 2.2				
		Bravecto^®^	15.8 ± 2.7	0.5(10.4) 0.616	0	− 0.8(8.1) 0.422	14.0	17.4 ± 2.6	0.6(10.0) 0.530	0	− 0.6(7.9) 0.590	7.9
		Simparica TRIO^®^	18.4 ± 1.8	1.4(8.0) 0.186	0			18.9 ± 1.5	1.6(8.2) 0.146	0		
	8 h	Untreated control	17.0 ± 2.6					18.1 ± 2.1				
		Bravecto^®^	4.4 ± 1.5	− 3.8(10.3) 0.003	74.0	− 4.4(8.0) 0.002	76.5	6.1 ± 2.0	− 4.2(13.9) < 0.001	66.2	− 5.2(13.3) < 0.001	68.2
		Simparica TRIO^®^	18.8 ± 1.5	0.6(10.5) 0.573	0			19.2 ± 1.6	0.4(13.1) 0.670	0		
	12 h	Untreated control	23.3 ± 2.9					24.4 ± 2.5				
		Bravecto^®^	0.2 ± 0.1	− 23(8.5) < 0.001	99.2	− 12(7.5) < 0.001	98.7	0.2 ± 0.2	− 9.7(7.0) < 0.001	99.0	− 5.6(7.0) < 0.001	98.5
		Simparica TRIO^®^	14.1 ± 3.1	− 2.2(8.9) 0.055	39.4			16.4 ± 2.9	− 2.1(13.7) 0.056	32.8		
	24 h	Untreated control	25.7 ± 2.6					26.5 ± 2.5				
		Bravecto^®^	0.0 ± 0.0	− 34(7.1) < 0.001	100	− 1.8(7.0) 0.107	100	− 0.0 ± 0.2	− 11(7.0) < 0.001	100	− 1.6(7.2) 0.157	100
		Simparica TRIO^®^	0.6 ± 0.4	− 11(9.1) < 0.001	97.8			1.0 ± 0.6	− 10(7.9) < 0.001	96.2		
Day 28 (28 days post-treatment)	4 h	Untreated control	15.0 ± 2.4					16.1 ± 2.2				
		Bravecto^®^	12.2 ± 1.6	− 1.0(5.3) 0.359	18.4	− 0.9(11.4) 0.376	19.6	12.9 ± 1.6	− 1.2(5.7) 0.271	20.2	− 1.2(10.1) 0.251	24.8
		Simparica TRIO^®^	15.2 ± 3.0	0.1(11.2) 0.954	0			17.1 ± 3.1	0.3(11.0) 0.797	0		
	8 h	Untreated control	18.1 ± 2.0					18.8 ± 1.8				
		Bravecto^®^	1.4 ± 0.6	− 7.3(9.1) < 0.001	92.2	− 7.1(10.5) < 0.001	92.5	2.1 ± 0.9	− 8.3(10.5) < 0.001	88.7	− 6.7(8.9) < 0.001	89.4
		Simparica TRIO^®^	18.9 ± 2.8	0.2(12.9) 0.814	0			20.1 ± 2.5	0.4(12.6) 0.662	0		
	12 h	Untreated control	23.9 ± 2.5					24.8 ± 2.2				
		Bravecto^®^	0.1 ± 0.1	− 29(9.3) < 0.001	99.6	− 17(7.9) < 0.001	99.5	0.1 ± 0.2	− 11(7.0) < 0.001	99.5	− 6.0(7.0) < 0.001	99.4
		Simparica TRIO^®^	17.3 ± 3.0	− 1.8(4.1) 0.138	27.7			19.3 ± 3.2	− 1.4(12.5) 0.179	22.2		
	24 h	Untreated control	27.9 ± 2.2					28.5 ± 2.2				
		Bravecto^®^	− 0.0 ± 0.0	− 45(7.2) < 0.001	100	− 2.4(7.0) 0.050	100	− 0.0 ± 0.2	− 13(7.0) < 0.001	100	− 2.0(7.1) 0.083	100
		Simparica TRIO^®^	1.0 ± 0.6	− 9.2(8.0) < 0.001	96.6			1.6 ± 0.8	− 11(8.8) < 0.001	94.3		

^1^Infestaton day relative to treatment day (Day 0)

^2^Tick count time post-treatment (Day 0) or post-reinfestation (Day 21 or 28)

^3^Any caculated negative efficacy values are reported as 0

**Fig. 1:**
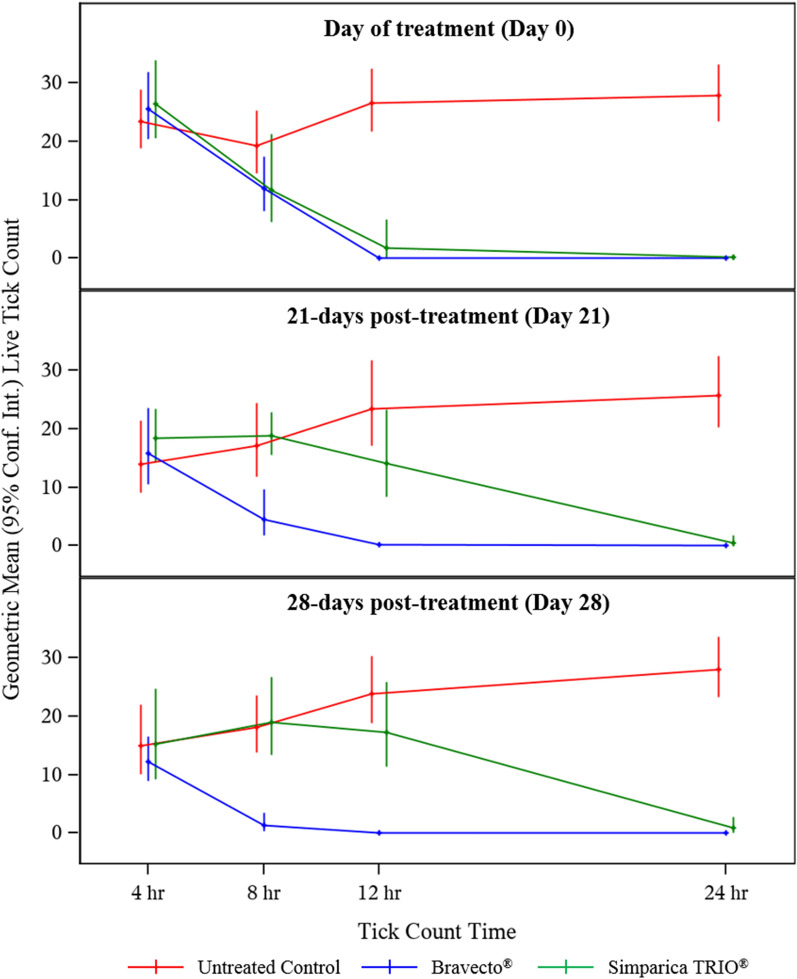
Tick kill rate of Bravecto^®^ and Simparica TRIO^®^ against adult *Ixodes scapularis* post-treatment and post-reinfestation. Live tick counts were performed on dogs treated with Bravecto^®^ or Simparica TRIO^®^ or untreated at 4, 8, 12 and 24 h post-treatment (Day 0) and post-reinfestation 21 and 28 days post-treatment. Geometric mean (95% confidence interval) live tick counts are displayed

Tick counts on untreated control dogs were adequate throughout the entire study (Table 1, Additional file 1: Figure S1). For initial speed of tick kill to control an existing *I. scapularis* infestation, while no efficacy was noted with either product 4 h post-treatment, significant efficacy was first noted for Bravecto^®^-treated dogs by 8 h post-treatment (*p* = 0.004) and by 12 h post-treatment for Simparica TRIO^®^-treated dogs (*p* = 0.001) (Table 2). By 12 h post-treatment, the tick control efficacy was 99.7% and 93.0% for Bravecto^®^- and Simparica TRIO^®^-treated dogs, respectively (Table 2, Fig. 1). By 24 h post-treatment, the efficacy of Bravecto^®^ and Simparica TRIO^®^ was 100% and 99.5%, respectively (Table 2; Fig. 1). All dogs in both groups had at least one live tick attached at 4 and 8 h post-treatment, but by 12 h post-treatment, 87.5% of the dogs in the Bravecto^®^ treatment group were tick-free, and by 24 h post-treatment, 100% of the dogs in the Bravecto^®^ treatment group were tick-free (Table 1).

For residual speed of tick kill efficacy at 21 and 28 days post-treatment against newly acquired ticks, Bravecto^®^-treated dogs had significantly fewer ticks at both the 8- and 12-h tick counts compared to both untreated control and Simparica TRIO^®^-treated dogs (Table 2, Fig. 1). Following tick infestation on Day 21, the efficacy at 8 and 12 h post-infestation was 74.0% and 99.2% for Bravecto^®^-treated dogs and 0.0% and 39.4% for Simparica TRIO^®^-treated dogs (Table 2). In contrast, over 90% tick control efficacy was not reached until 24 h post-Day 21 infestation (efficacy = 97.8%) for the Simparica TRIO^®^ treatment group (Table 2).

Following tick infestations on Day 28, the efficacy for Bravecto^®^- and Simparica TRIO^®^-treated dogs at 8 h post-infestation was significantly higher for Bravecto^®^-treated dogs (92.2% vs. 0.0%, *p* ≤ 0.001) (Table 2, Fig. 1). By 12-h post-Day 28 infestation, efficacy remained significantly greater for Bravecto^®^- compared to Simparica TRIO^®^-treated dogs (99.6% vs. 27.7%, *p* ≤ 0.001) (Table 2). By 24-h post-Day 28 infestation, the tick control efficacy was 100% and 96.6% for Bravecto^®^ and Simparica TRIO^®^ treatment groups, respectively (Table 2).

Following the Day 21 infestation, 75.0% and 100% of dogs in the Bravecto^®^ treatment group were tick-free (i.e. free of live ticks) at 12 and 24 h post-infestation, respectively, whereas in the Simparica TRIO^®^ group, none of the dogs were tick-free at 12 h post-infestation and 62.5% were tick-free at 24 h post-infestation (Table 1). Similarly, following the Day 28 infestation, 87.5% and 100% of dogs were tick-free in the Bravecto^®^ treatment group by 12 and 24 h post-infestation, respectively. In contrast, following the Day 28 infestation, none of the dogs in the Simparica TRIO^®^ group were tick-free at 12 h post-infestation and 50.0% were tick-free at 24 h post-infestation (Table 1). Significantly more dogs were tick-free by 12 h post-infestation in the Bravecto^®^ treatment group on Day 21 (75% vs. 0%, *p* = 0.007) and Day 28 (87.5% vs. 0%, *p* = 0.001) compared to the Simparica TRIO^®^ group.

## Discussion

This efficacy study provides a detailed evaluation of Bravecto^®^ efficacy against *I. scapularis* infesting dogs at multiple time points within 24 h post-treatment or post-infestation during the first 30 days of treatment. When evaluating the initial speed of tick kill (i.e. how quickly a product kills ticks already attached upon treatment), it was determined that a single oral treatment of Bravecto^®^ reduced live adult *Ixodes scapularis* counts by 99.7% within 12 h of treatment and by 100% at 24 h post-treatment. The initial speed of kill observed in this study is similar to the efficacy previously reported for Bravecto^®^ against *Ixodes ricinus*-infested dogs [19]. It that study, efficacy at 12 and 24 h post-treatment was 97.9% and 100%, respectively.

In this study, the initial 24-h efficacy of Bravecto^®^ (100%) was similar to that of Simparica TRIO^®^ (99.5%) (Table 2). The Simparica TRIO^®^ results are similar to those of a previous study that demonstrated 99.4% efficacy against *I. scapularis* at 24 h post-treatment [24].

Evaluation of the residual speed of tick kill is important when trying to assess the ability of an acaricide to prevent or minimize pathogen transmission. Tick-borne pathogen transmission timing is variable and pathogen dependent [25–27]. A prevailing theme is that *Ixodes* ticks must be attached and feeding for about 24 to 48 h before pathogen transmission occurs, and this is generally the case for transmission of *B. burgdorferi* and *A. phagocytophilum* by *I. scapularis* [25–31]. A review of studies investigating *B. burgdorferi* transmission timing by a single infected tick reported no findings of transmission within 24 h of infestation, ~ 10% transmission within 48 h of infestation, 70% transmission within 72 h of infestation and 90% transmission by completion of tick feeding at approximately 7–9 days [26]. Transmission timing data for other tick-transmitted pathogens is sparse, but, in general, tick-borne viral pathogens are transmitted most quickly (minutes to hours of tick attachment), tick-borne bacterial pathogens are transmitted within hours to a few days of tick attachment (tick-borne pathogen dependent), and tick-borne protozoal pathogens are transmitted the slowest (multiple days after tick attachment) [25, 26, 32, 33]. However, transmission timing may be influenced by additional factors that can speed up or slow down transmission time, including co-feeding by multiple infected ticks, tick or pathogen genetics, the influence of co-occurring microbes and interrupted tick feeding followed by reattachment to a naïve host [26, 29, 34, 35].

A single treatment of Bravecto^®^ significantly reduced live adult *I. scapularis* counts by 8 h  post-treatment and post-infestion at 21 days and 28 days post-treatment compared to untreated controls (38.0%, 74.0% and 92.2%, respectively) (Table 2). Efficacy of Bravecto^®^ was > 99% at 12 h post-treatment and post-infestation at 21 or 28 days post-treatment. At both 21 days and 28 days post-treatment, Bravecto^®^ significantly outperformed Simparica TRIO^®^ at 8- and 12-h post-infestation time points, with fewer live ticks identified on Bravecto^®^-treated dogs. The efficacy of Simparica TRIO^®^ at 8 and 12 h on Day 28 was only 0% and 27.7%, respectively. Similar to a previous study, a slower residual speed of tick kill efficacy was observed for Simparica TRIO^®^-treated dogs, where the efficacy of Simparica TRIO^®^ against *I. scapularis* at 8 and 12 h post-infestation on 28 days post-treatment was 3.1%, and 52.2%, respectively [24].

The residual efficacy of Simparica TRIO^®^ against *I. scapularis* observed in this and the previously mentioned study [24] is contrasted by the residual efficacy observed in studies evaluating a stand-alone oral sarolaner product (Simparica^®^). In one study, when oral sarolaner was administered at 2–4 mg/kg, residual efficacy against *I. scapularis* 21 days post-treatment was 33.5% and 68.8%, respectively, at 8 and 12 h post-infestation [36]. In the current study, efficacy at 8 and 12 h post-infestation 21 days post-treatment were 0.0% and 39.4%, respectively (Table 2). Additionally, in another stand-alone oral sarolaner study, efficacy at 12 h after reinfestation 28 days post-treatment was 62.0% [37], much greater than the 27.7% efficacy observed in this study at the same time period. The minimal prescribed dose of sarolaner in the stand-alone product (Simparica^®^) is 2 mg/kg, a 40% higher minimum dose than the combination product, Simparica TRIO^®^ (1.2 mg/kg). Dogs in this current study received a range of 1.6–2.3 mg/kg sarolaner, which is within the prescribed dose range for Simparica TRIO^®^, the sarolaner product used in this study.

A greater percentage of tick-free dogs were observed among Bravecto^®^-treated dogs 21 days post-treatment, with 75.0% (6/8) and 100% (8/8) dogs tick-free at 12 and 24 h post-infestation compared to 0% (0/8; *p* = 0.007) and 62.5% (5/8; *p* = 0.200) of the Simparica TRIO^®^-treated dogs at those time points (Table 1). Similarly, at 28 days post-treatment, 25% (2/8), 87.5% (7/8) and 100% (8/8) of the Bravecto^®^-treated dogs were tick-free by 8, 12 and 24 h post-infestation, respectively. However, following the 28-day post-treatment infestation, none of the Simparica TRIO^®^-treated dogs were tick-free until 24 h post-infestation (50%, 4/8) (Table 1).

Although both products effectively killed adult *I. scapularis*-infested dogs by 24 h post-treatment (initial speed of kill) or post-infestation 21 and 28 days post-treatment (residual speed of kill), dead ticks attached to dogs were often observed at these time points. These dead attached ticks were clearly dead (i.e. no movement to stimuli, shriveled and desiccated, missing legs due to desiccation). As ticks effectively ‘glue’ their mouthparts to the skin of their host, reinforced by barbs decorating their mouthparts that anchor them into the skin, it can take awhile to fully dislodge a dead tick that is still physically associated to the host. As an active feeding process is required to pump tick-borne pathogens from the salivary glands via the saliva into the host, a dead attached tick poses no tick-borne pathogen transmission risk to the host. Ticks with longer mouthparts such as *I. scapularis* and *Amblyomma* spp. are more likely to be found still attached, even after they are dead, on dogs treated with systemically acting isoxazoline tick control products. Because ticks must engage in the attachment/feeding process to come into contact with these drugs, observation of dead attached ticks can occur.

## Conclusions

*Ixodes scapularis* infestations are controlled more quickly 21 and 28 days post-dosing on dogs administered a single treatment of Bravecto^®^ compared to dogs administered a single treatment of Simparica TRIO^®^. Additionally, 100% of dogs treated with Bravecto^®^ were free of live ticks by 24 h post-treatment and by 24 h post-infestation at 21 and 28 days post-treatment.

A single oral treatment of either Bravecto^®^ or Simparica^®^ TRIO effectively reduced live tick counts by > 94% by 24 h post-treatment or infestation, both within their FDA-approved label indications. The efficacy (reduction in established live tick counts) of both products are similar upon initial administration. However, likely due to the reduced amount of sarolaner in Simparica TRIO^®^, ticks are killed significantly slower at 21 and 28 days in dogs administered Simparica TRIO^®^ compared with Bravecto^®^-treated dogs. Use of tick control products that kill ticks quickly is important to reduce the risk of tick-borne pathogen transmission to dogs.

### Supplementary Information

**Additional file 1: Fig. S1**. Plots of live tick counts over time by individual dogs within the same block. Dogs were arranged in blocks after test infestation by descending live tick counts. Dogs within a block were randomly allocated to treatment groups. Live tick counts are presented for individual dogs, organized by block, at 4, 8, 12 and 24 h post-treatment (Day 0) and post-reinfestation 21 and 28 days post-treatment.

## Data Availability

All relevant data generated or analyzed during this study are included in this published article. The summary data used and analyzed are available upon reasonable request from the corresponding author.
