# Supplementary figures and images for: Comparison of the initial and residual speed of Ixodes scapularis kill on dogs treated with a single dose of Bravecto® Chew (25 mg/kg fluralaner) or Simparica TRIO® (1.2 mg/kg sarolaner, 24 µg/kg moxidectin, 5 mg/kg pyrantel)
Source: Parasit Vectors. 2023 Nov 27;16:440. doi: 10.1186/s13071-023-05946-3 (PMC10683217; doi:10.1186/s13071-023-05946-3)

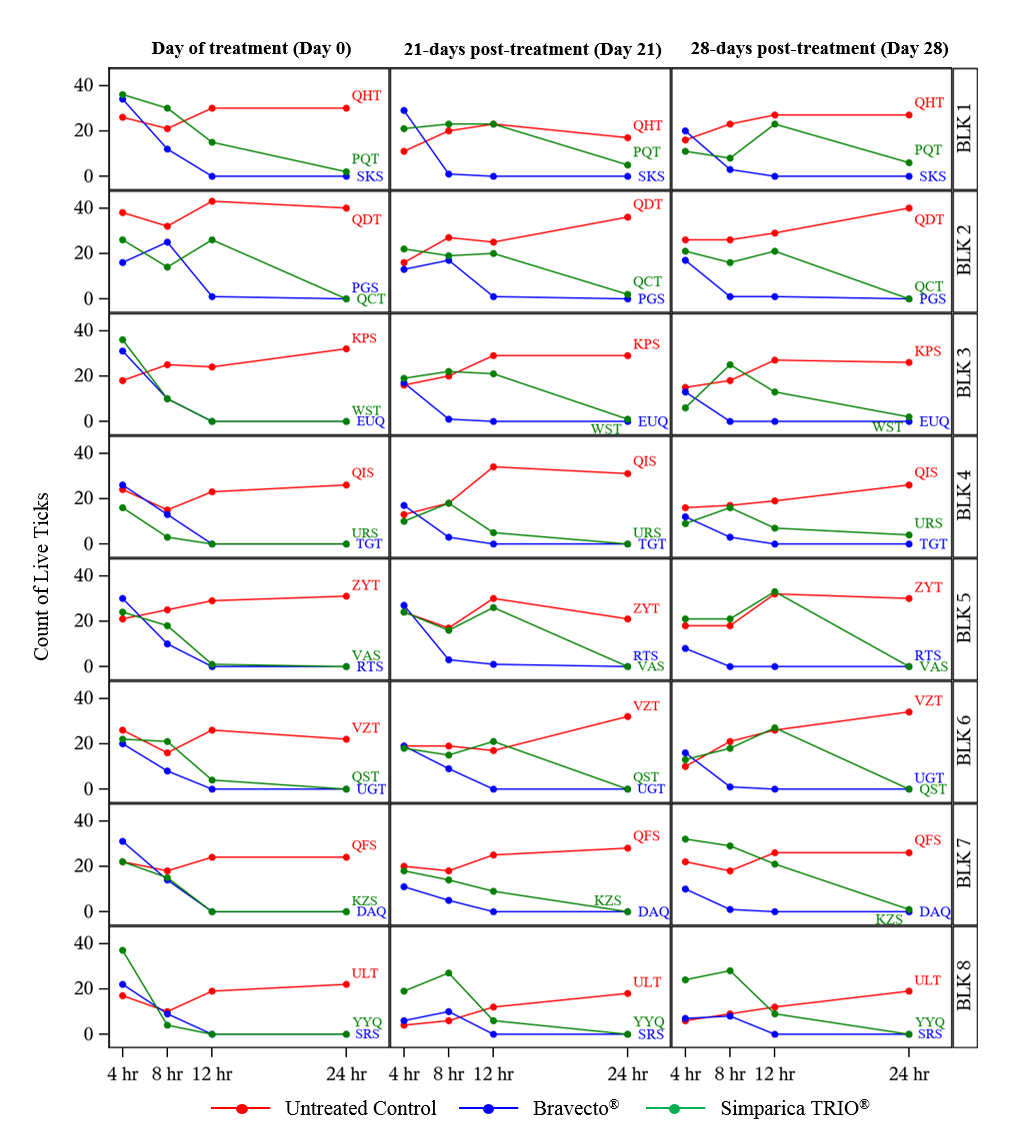

Supplement: Supplementary file 1 — Additional file 1: Fig. S1. Plots of live tick counts over time by individual dogs within the same block. Dogs were arranged in blocks after test infestation by descending live tick counts. Dogs within a block were randomly allocated to treatment groups. Live tick counts are presented for individual dogs, organized by block, at 4, 8, 12 and 24 h post-treatment (Day 0) and post-reinfestation 21 and 28 days post-treatment. [file 13071_2023_5946_MOESM1_ESM.png]
